# Access to specialty care in autism spectrum disorders-a pilot study of referral source

**DOI:** 10.1186/1472-6963-11-99

**Published:** 2011-05-14

**Authors:** Xue Ming, Anjum Hashim, Sharon Fleishman, Therese West, Ning Kang, Xiang Chen, Barbie Zimmerman-Bier

**Affiliations:** 1 Department of Neurosciences and Neurology, UMDNJ-New Jersey Medical School, 185 South Orange Avenue, Newark, NJ, USA; 2 The Autism Center, UMDNJ-New Jersey Medical School, 185 South Orange Avenue, Newark, NJ, USA; 3 Department of Pediatrics, Newark Beth Israel Hospital, 211 Lyons Avenue, Newark, NJ, USA; 4 Health Benchmarks Inc., IMS Health, 21650 Oxnard street, Woodland Hills, CA, USA; 5 Department of Neurology, Yu-Yin Children's Hospital, WenZhou Medical College, Wenzhou, China; 6 Department of Pediatrics, Saint Peter's University Hospital, 254 Easton Avenue, New Brunswick, NJ, USA

## Abstract

**Background:**

In the United States, a medical home model has been shown to improve the outcomes for children with special health care needs. As part of this model, primary care physicians provide comprehensive medical care that includes identification of delayed and/or atypical development in children and coordination of care with specialists. However, it is not clear if families of children with Autism Spectrum Disorder (ASD) rely on the medical home model for care of their child to the same extent as families of children with other special health care needs. This study aims to add to the understanding of medical care for children with ASD by examining the referral source for specialty care.

**Methods:**

This retrospective study was accomplished by evaluating parent completed intake data for children with ASD compared to those with other neurological disorders in a single physician Pediatric Neurology Practice at a major urban medical center in Northern New Jersey. To account for referral bias, a similar comparison study was conducted using a multispecialty ASD practice at the same medical center. Parent reported "source of referral" and "reason for the referral" of 189 ASD children and 108 non-ASD neurological disordered children were analyzed.

**Results:**

The specialty evaluations of ASD were predominantly parent initiated. There were significantly less referrals received from primary care physicians for children with ASD compared to children with other neurodevelopmental disorders. Requirement of an insurance referral was not associated with a primary care physician prompted specialty visit.We identified different patterns of referral to our specialty clinics for children with ASD vs. children with other neurolodevelopmental disorders.

**Conclusion:**

The majority of the families of children with ASD evaluated at our autism center did not indicate that a primary care physician initiated the specialty referral. This study suggests that families of children with ASD interface differently with the primary care provider than families of children with other neurological disorders.

## Background

Early detection and treatment of developmental disorders such as Autism Spectrum Disorders (ASD) is crucial in order to maximize developmental outcomes, assist with family planning and prevent secondary co-morbidities. Recent evidence shows that early intensive behavior interventions in children diagnosed with ASD prior to age 3 can result in significant improvement in cognitive, social and adaptive functioning in children with ASD [1-3]. Pediatricians and other primary health care physicians may play a pivotal role in the early detection of ASD because they provide developmental surveillance and screening for all children as part of a medical home [4,5]. Specialty referral is often necessary to confirm a diagnosis of ASD and evaluate medical co-morbidities [6]. In the United States, pediatricians coordinate referrals to a number of specialists including developmental pediatricians, child neurologists, psychologists, geneticists, gastroenterologists, allergists/immunologists, and child psychiatrists as well as early intervention and school programs as part of medical home for children with ASD. However, families may initiate a specialty consultation due to concerns arising between scheduled well care visits or at the advice of family and friends. These consultations with specialists could occur outside of the medical home.

ASD are not rare and many primary care pediatricians need to provide comprehensive care for children with ASD. Recent statistics provided by the Center for Disease Control and Prevention indicate that the prevalence of ASD is one in 94 [7]. However, it has been recognized that some primary care pediatricians may hold outdated beliefs about ASD, do not routinely screen for ASD and may be unaware of resources for diagnosis and treatment for ASD [6]. In an effort to improve early detection of ASD, a number of recent public service initiatives and professional resources have been developed to improve recognition of ASD in the home, school and primary care medical practice. Other efforts to improve detection and care for children with ASD have included updated practice parameters [5,6] addressing screening, surveillance, and treatment for ASD in the medical home.

The medical home model for children with special health care needs should not differ by diagnosis, yet our anecdotal experience showed that families of ASD tend to access specialty care by self referral rather than relying on their primary care physicians' recommendation. Brachlow et al. [8] stated "a large percentage of children with autism do not receive primary care consistent with that in a medical home". Our study focused on understanding the source of referral for specialty consultation through the families' perspective. It is important to understand the families' perspective in order to ensure that a medical home is comprehensive, continuous, coordinated and responsive to the needs of the family. To determine whether ASD families relied on their primary care physicians in initiating the specialty assessment of this disorder, we compared the referral patterns from the perspective of the family, of ASD children with those of other neurological disorders.

## Methods

A retrospective chart review of both electronic and paper records between April 2005 and June 2006 was performed to compare the referral source of ASD children and non-ASD children evaluated by a single practitioner (pediatric neurology) practice (XM), and the referral sources of ASD children evaluated by specialists in a multispecialty autism center.

This study was approved by the Institutional Review Board of the New Jersey Medical School, University of Medicine and Dentistry of New Jersey. Informed consent was waived due to the retrospective nature of this study.

### Subjects and Settings

Our medical center is located in an economically disadvantaged community and provides a substantial amount of specialty care. The Autism Center is an independent multispecialty practice provided by specialists from different departments of the medical center. The 108 families of children with a diagnosis of ASD were selected from the practice of XM, a pediatric neurologist at the Autism Center. An age-matched comparison group consisted of first 108 consecutive children with non-autistic neurological disorders. These children were evaluated by XM at the general Pediatric Neurology clinic of the same institution during the same time period. These families were studied because a similar intake form was used that detailed the referral source in both clinical settings (Autism Center vs Pediatric Neurology clinic of XM). To avoid physician referral bias we also compared the referral sources of ASD children evaluated by the pediatric neurologist and by those of other specialists of the Autism Center. This allowed us to determine whether the referral patterns were similar across specialties and physicians.

There were 213 patients evaluated in the Autism Center during this time period and 189 children were diagnosed as ASD. All 189 children had confirmed diagnosis of ASD based on DSM IV criteria; 97 children were diagnosed previously and confirmed during the evaluation of this study. Of the 189 children with ASD, 108 children were evaluated by XM. The remaining 81 children with ASD were evaluated by other specialists of the Autism Center. The specialty services available at the Autism Center included pediatric neurology, developmental pediatrics, child psychiatry, psychology, medical genetics, pediatric gastroenterology, immunology and allergy etc.

### Data Acquisition

Information extracted from the initial intake forms, registration, billing, and chart records included: participant's date of birth, gender, source of referral, type of health insurance, if the insurance required a physician referral for the specialist visit, diagnosis and comorbidities. The intake forms listed demographic, insurance and pediatrician/primary care physician's contact information, referral source, and a detailed description of reasons for evaluation. For ASD patients, the parents were asked to check off pertinent medical and behavioral problems from a list and provide a brief written description, document developmental milestones (including regression). The source (who initiated the specialty visit) was provided as a checklist for parents to complete. Parents could indicate if the source of the referral was the pediatrician/primary care physician; parents; family members or friends; school teacher/therapist; or others (specify). Completed intake forms were reviewed by the advanced nurse practitioner (TW) and the developmental pediatrician (BZB) who assigned the patient to a specialist in the Autism Center. Specific specialty consultations could also be requested by the family or primary care physician The criteria for assignment to specialists was based on several referral categories: 1) neurological evaluation for epilepsy, tic disorder or movement disorder, sleep disorders, attention deficit hyperactivity disorders, gross or fine motor delay, and/or developmental regression; 2) developmental pediatric evaluation for patients whose parents listed the reason for evaluation as confirmation of diagnosis, speech and language delay, school performance problems and other developmental delay/concerns; 3) psychiatric or psychological/behavioral evaluation for patients with aggression, irritability, and extreme compulsive behaviors. If the parents indicated that psychotropic medication use was necessary, the patient was assigned to a child psychiatry evaluation; 4) medical genetic evaluation when the referral indicated a need for a genetic workup; 5) gastroenterological evaluation if gastrointestinal problems were the major concern for the visit; 6) allergy/immunological evaluation if the parents listed allergy for the reason of the visit. The intake forms were completed by the parents and sent to the clinic/center prior to scheduling. Once the patient was assigned to a specialist and an appointment was made, the registration staff coordinated the necessary insurance precertification and primary care documentation needed for the visit.

Sources of referral were then divided into two groups; (a) physicians, (b) non-physicians. The non-physician group consisted of parents or friends of parents, school officials or therapists, and others (insurance providers and child advocates). Medical insurer was defined as either private/commercial insurance or Medicaid/charity care. The type of insurance was used as a surrogate of socioeconomic status (SES), with Medicaid or charity care considered as lower SES and private/commercial insurance regarded as higher SES. The reason for the referral was divided into disorders commonly perceived as medical/physical and those viewed as behavioral/developmental disorders. The medical/physical disorders included epilepsy, headache/migraine, syncope, hydrocephalus, brain tumor, head trauma, movement disorders, craniofacial malformation, neuromuscular disorders, sleep disorders, microcephaly, cerebral palsy, gastrointestinal disorders (such as chronic constipation, reflux etc), allergy, food intolerance, circulatory disorders, cerebral vascular disease, illicit drug exposure, or known infectious diseases etc. The behavioral or developmental disorders consisted of attention deficit hyperactivity disorder, mood disorders, anxiety disorder, oppositional defiant disorder, aggression, speech and language delay or disorder, general developmental delay, learning disability etc.

### Data Analysis

The data was extracted and input into an Excel database. Variables of each category were coded and tallied. A statistical program SAS version 9.2 was used for statistical analysis. The referral source of the ASD children and non-ASD children evaluated by XM were compared. The referral source of ASD children evaluated by XM and the other members of the Autism Center were also compared. 2 × 2 or 2 × 4 Chi-square tests were used for statistical analysis.

## Results

The demographic characteristics of the ASD children and controls are shown in Table 1. There is a high ratio of male to female in ASD, consistent with the male dominance of this disorder. There were significantly more children with ASD than children in the comparison group who had a private insurance plan (p < 0.001), suggesting that our clinic families of children with ASD may have higher SES.

**Table 1:** Demographic characteristics and referral pattern

**Groups**^**1**^		ASD/XM n, (%)	ASD/TAC n, (%)	Comparison n, (%)
**Age in years (range, median, mean ± SD)**		3-19, 8, 8.1 ± 4.1	2-18, 6.5, 6.6 ± 4.1	0.5-18, 8.5, 8.4 ± 4.9

**Male: Female**		86:22**	61:20*	66:42

**Insurance Provider**	*Private*	56 (52%)***	53 (65%)***	18 (17%)
	
	*Medicaid/Charity Care*	52 (48%)	28 (35%)	90 (83%)

**Referral source**	*Physicians*	33 (30%)***	31 (38%)***	89 (83%)
	
	*Self*	57 (53%)	39 (49%)	8 (7%)
	
	Therapists or Teachers	15 (14%)	6 (7%)	9 (8%)
	
	Other	3 (3%)	5 (6%)	2 (2%)

1. ASD/XM and Comparison denote children with ASD and other neurological disorders evaluated by XM respectively; ASD/TAC denotes the group of children with ASD evaluated by other members of The Autism Center.

2 × 2 Chi square tests were performed for gender and insurance providers between ASD/XM and Comparison, between ASD/TAC and Comparison. 4 × 2 Chi square tests were performed for referral source between ASD/XM group and Comparison group, and between ASD/TAC group and Comparison group.

*p < 0.05, **p < 0.01, ***p < 0.001.

The diagnosis of ASD was made based on the DSM-IV criteria and included autistic disorder (n = 85, 45%), pervasive developmental disorder-not otherwise specified (n = 93, 49%) and Asperger's disorder (n = 11, 6%). The diagnosis of non-autistic comparison children was made by the neurologist, including epilepsy (n = 23, 21%), cerebral palsy (n = 10, 9%), headache (n = 13, 12%), attention deficit hyperactivity disorder (n = 20, 18%), learning disability (n = 2, 2%), speech/language disorders and non-autistic developmental delay (n = 16, 15%), hydrocephalus (n = 5, 4%), head trauma (n = 5, 4%), movement disorders (n = 5, 4%), neuromuscular disorders (n = 4, 4%), syncope (n = 4, 4%), others (n = 14, 13% including macro- or microcephaly, sleep disorders, skull deformity, neurocutaneous disorders etc). A child may have multiple diagnoses. More than half of the children with ASD were privately insured and the remaining ASD children received Medicaid or Charity Care. Only a small portion of the children in the comparison group had private insurance and the majority was insured by Medicaid or Charity Care.

Within all the patients evaluated by the Pediatric Neurologist, 122 (56.6%) children were referred by another physician. Almost 93% of the physicians who initiated the referral were primary care physicians. The majority of the non-autistic children were referred by a physician (Table 1). However less than one third of the ASD children were referred by a physician. Other referral sources for these children with autism included; 53% (57) parents, and 17% (18) school, therapist or other referrals. In those without ASD, only 7% were parent initiated, the remaining 10% were school or therapist initiated assessment. Comparison of referral sources between ASD/XM and Comparison groups showed significant different patterns (p < 0.001). In contrast to Comparison group, there were more non-physician initiated referrals than physician initiated referrals for the ASD group (ASD/XM).

In order to account for possible referral biases a second study was completed. ASD patients evaluated by the pediatric neurologist (ASD/XM) were compared to the ASD patients seen at a multidisciplinary clinic (ASD/TAC). In ASD/TAC group, 31 (38%) of patients were referred by a physicians, compared to 50 (60%, sum of all non-physician sources) of patients whose referral was initiated by a non-physician source (Table 1). Comparison of the referral sources between ASD/TAC and Comparison group showed a significantly different patterns (p < 0.001). Similar to ASD/XM, this group of ASD children (ASD/TAC) had more non-physician sources of referral. Although there was less physician referral of ASD evaluated by XM (30%) than that of ASD patients evaluated by the other specialists in the Autism Center (38%), the difference was not statistically significant (p = 0.27). Similarly, there was no significant difference between the non-physician referral sources for the ASD patients evaluated by XM (70%) and of the other specialists in the Autism Center (62%, p = 0.27).

Many of the current managed health care plans require an insurance referral documents from primary care physicians to reimburse a specialist visit. Families of children with ASD in our study had higher SES that might enable them to choose a freedom access insurance plan. The freedom of access to specialty care may have contributed to the less physician initiated referral in ASD children. To determine whether this was a confounding factor, Chi-square tests were performed to evaluate whether the insurance requirement was associated with a physician initiated referral in each of the three groups (Table 2). The results showed no association between the insurance requirement and the referral source for the visit (p = 0.48 within ASD/XM group, p = 0.87 within Comparison group, p = 0.64 within ASD/TAC group), suggesting that the different patterns of referral source between the ASD groups and Comparison group were independent of insurance requirement.

**Table 2:** Sources of Referral and Health Insurance Requirement of Referral ^1^

		ASD/XM		ASD/TAC		Comparison	
		
		Physician	Non-physician	Physician	Non-physician	Physician	Non-physician
**Insurance Referral**	**Yes**	20	40	20	10	58	12
	
	**No**	13	35	27	17	31	7

**Chi Square Probability**		p = 0.48		p = 0.64		p = 0.87	

1. The requirement of a document provided by primary care physicians to specialist office visits among subjects referred for initial visit by physicians or non-physicians within each group.

The reasons for referral were evaluated to determine if they were potential contributory factors in generating physician initiated referrals. The determination of reasons for referral was based on the chief complaint checklists caregivers offered on the intake forms as reasons for the visit. The parental complaints of medical/physical problems in ASD included sleep disorders (n = 23), epilepsy (n = 11), gastrointestinal dysfunction (n = 31), allergy (n = 4), food intolerance (n = 11) and others (n = 5). The behavioral/developmental complaints these parents with ASD children offered were irritability (n = 45), compulsion (n = 59), aggressiveness (n = 34), anxiety (n = 31), sensory hypersensitivity (n = 27), speech and language delay (n = 124), motor incoordination (n = 24) etc. Many families indicated more than one concern. At least 74% of all ASD and 35% of non-autistic children were evaluated for developmental delay or behavioral disorders. In contrast, almost twice as many (65%) of non-autistic children were evaluated for medical concerns, while only approximately one fourth of the ASD children were referred for medical reasons. Of the ASD children referred for behavioral or developmental disorders, slightly over one fourth of them were referred by physicians, while approximately half of the ASD children whose reason for referral was the presence of medical/physical disorder(s) were referred by physicians (Table 3). The chief complaint of nine ASD children included both medical and behavioral disorders; four of these nine ASD children were referred by a physician. The caregiver of one ASD child did not offer a chief complaint. This child was referred by a physician. Of the 38 non-autistic children with behavioral or developmental disorders, 25 (28%) of them were referred by physicians. Of the 70 non-autistic children whose chief complaints were medical/physical disorders the majority (72%) were referred by physicians. In ASD groups, behavioral or developmental problems appeared to be the predominant reasons for referral by both physicians and non-physicians. In the Comparison group, the reasons for referral by physicians were mainly medical or physical disorders, while those by non-physicians were more likely behavioral/developmental.

**Table 3:** Reasons and Sources of Referral

Reason for Referral	**ASD/XM**^**1**^			**ASD/TAC**^**1**^			**Comparison**^**1**^		
	
	Total	Physician	Non-physician	**Total**^**2**^	Physician	Non-physician	Total	Physician	Non-physician
**Behavioral/Developmental**^3^	84	21 (64%)	63 (84%)	56	18 (62%)	38 (74%)	38	25 (28%)	13 (68%)

**Medical**^3^	24	12 (36%)	12 (16%)	15	7 (24%)	8 (16%)	70	64 (72%)	6 (32%)

**Medical and Behavioral/Developmental**^3^	0	0	0	9	4 (14%)	5 (10%)	0	0	0

1. ASD/XM and Comparison denote children with ASD and other neurological disorders evaluated by XM respectively; ASD/TAC denotes the group of children with ASD evaluated by other members of The Autism Center.

2. One ASD whose chief complaint was not offered is not included. See text.

3. The numbers represent the number of children in each category. The numbers in parenthesis represent the percentage of total in each category.

## Discussion

This study evaluated the referral pattern of ASD to specialty care in our Autism Center. Our results showed that, in contrast to non-autistic patients, the majority of the ASD children's specialist evaluations were reportedly non-physician initiated. The reasons for referral in ASD children were predominantly developmental delay or behavioral disorders.

The reason(s) for these differences in referral source for specialist evaluation for children with ASD in our Autism Center is not clear. The reason(s) families of children with ASD initiated specialty evaluations without involvement of their primary care physicians remain to be determined. Health insurance plans did not appear to affect the referral sources within our cohort. We did not systematically determine what ASD families did in their treatment seeking process. It is possible that with the widely available medical information in the internet, parents may choose to bypass the cumbersome health care system and generate a specialty evaluation without going through primary care physicians. Extensive outreach strategies in the community, the addition of public health initiatives to increase awareness of ASD, and the impact of family and friends may have directly increased ASD specific self referral into the Autism Center. Another possibility could be that medical disorders (including neurological disorders) in ASD are under-recognized in general, resulting in the lower referral rate by primary care physicians. It is also possible that current managed health care system with complicated referral process and the limited time to interface with parents may have contributed to the lower physician referral to specialists for ASD. Finally, while the American Academy of Pediatrics recommendations released in 2001 [5], and revised in 2007 [6], indicate that pediatricians should screen for ASD and perform diagnostic evaluations, some pediatric practices may have not yet implemented these guidelines during the study time. We are currently underway to perform a follow-up to this initial study.

The nature of symptoms did not appear to be a factor in generating the referral bias for ASD in this study. If the argument that the symptoms of children with ASD were predominantly behavioral or developmental and these symptoms were a factor contributing to less referral by physicians, then such a bias should apply to non-autistic children with developmental or behavioral symptoms as well. However, this study showed that non-autistic children with developmental delay or behavioral disorders were predominantly referred by a physician. This study included referral to multiple specialties including developmental pediatrician, pediatric neurologist, psychiatrist, psychologist, thus reducing the referral bias that may be associated with the results.

This preliminary study aims to begin examining relationships between primary care pediatricians and specialists in terms of disease specific referrals. We believe that primary care physicians are integral members of the team of physicians who care for children with ASD. Although it is beyond the scope of this study, one fundamental question that remains is whether these differences in referral patterns may underlie differences in primary care for children with ASD and if care will be improved by ensuring that the primary care medical home model is widely utilized. Changes in access to specialty care may lead to different health care outcomes. Pediatricians perform preventive services not found in specialty clinics. Parents may not be aware of the differences in care in primary vs. specialty setting and ultimately fail to obtain health care screenings and routine care. In addition, health care costs may be increased if care is not coordinated. If health care systems are not addressing primary health care needs of ASD patients then those systems may need to change. For example, there currently is no provision for a well-child care visit between the ages of 2 and 3, a period when some ASD specific features become apparent. This may lead to parents erroneously feel pediatricians may have missed an ASD diagnosis.

There were potential confounding factors to keep in mind when interpreting the results. Our database suggests that the families of ASD came from a diverse geographic area that can be distant from the location of our hospital, while the non-autistic children were largely from the local urban area. The larger geographic area of residents of the ASD children could contribute to the lower physician generated referral. Furthermore, pediatricians are capable of treating ASD by referring to interventional therapists without the need of involving another physician. This could potentially contribute in part to the lower referral rate for ASD. Another potential confounding factor is that this study was limited by information provided by the family. We could not ascertain if the pediatricians referred the family elsewhere, or families initiated specialty care elsewhere due to our facility not participating in their insurance plans. Future studies on primary care referral patterns for children with ASD and other neurodevelopmental disabilities are needed. In addition, similar studies in other regions of the country will provide information as to whether this referral pattern is local or widespread.

## Conclusions

The majority of the families of children with ASD evaluated at our autism center did not indicate that a primary care physician had initiated the specialty referral. Our study suggests that families of children with ASD initiate medical consultation with specialists more than families of children with other neurodevelopmental disorders.

## Competing interests

The authors declare that they have no competing interests.

## Authors' contributions

XM: carried out the study design, Institutional Review Board application; coordinated and supervised the study personnel, participated in data collection, wrote and submitted the manuscript. AH, TW and XC participated in data collection; FS participated in manuscript writing, data verification and organization; NK: performed statistical analysis; BZB participated in data interpretation and manuscript writing.

All authors have read and approved the final manuscript.

## Pre-publication history

The pre-publication history for this paper can be accessed here:

http://www.biomedcentral.com/1472-6963/11/99/prepub
